# AI-engineered multifunctional nanoplatforms: synergistically bridging precision diagnosis and intelligent therapy in next-generation oncology

**DOI:** 10.1186/s12951-025-03947-1

**Published:** 2025-12-20

**Authors:** Lin Zhao, Xinglong Liu, Xiangying Deng

**Affiliations:** 1 Department of Pathology, The Second Xiangya Hospital, Central South University, Changsha, 41001l Hunan China; 2 Hunan Clinical Medical Research Center for Cancer Pathogenic Genes Testing and Diagnosis, Changsha, 410011 Human China; 3 Institute of Medical Sciences, National Clinical Research Center for Geriatric Disorders, Xiangya Hospital, Central South University, Changsha, 410008 Hunan China

**Keywords:** Artificial intelligence, Nanotechnology, Intelligent nanoplatforms, Personalized medicine, Translational challenges

## Abstract

The integration of artificial intelligence (AI) and nanotechnology is reshaping cancer diagnosis and treatment. In this context, intelligent nanoplatforms are multifunctional nanoscale systems designed or optimized with the help of AI, capable of combining tumor sensing, targeted delivery, controlled release, and adaptive response within a single platform. AI can analyze large-scale multi-omics and clinical datasets to support early cancer detection, accurate diagnosis, prognosis assessment, and refinement of personalized treatment strategies, while nanotechnology enables precise tumor targeting and site-specific drug delivery through diverse nanocarriers, thereby reducing systemic toxicity and improving therapeutic efficacy. Their interaction allows more rational nanomedicine design by optimizing key properties such as targeting capability, stability, and responsiveness, and nano-enabled imaging and sensing provide high-resolution data that further enhance model performance. Together, these advances point toward more personalized and efficient strategies for cancer diagnosis, therapy, and monitoring, although challenges related to data sharing, standardization, privacy, ethics, regulation, and development costs still need to be addressed for broader and safer clinical implementation.

**Graphical abstract:**

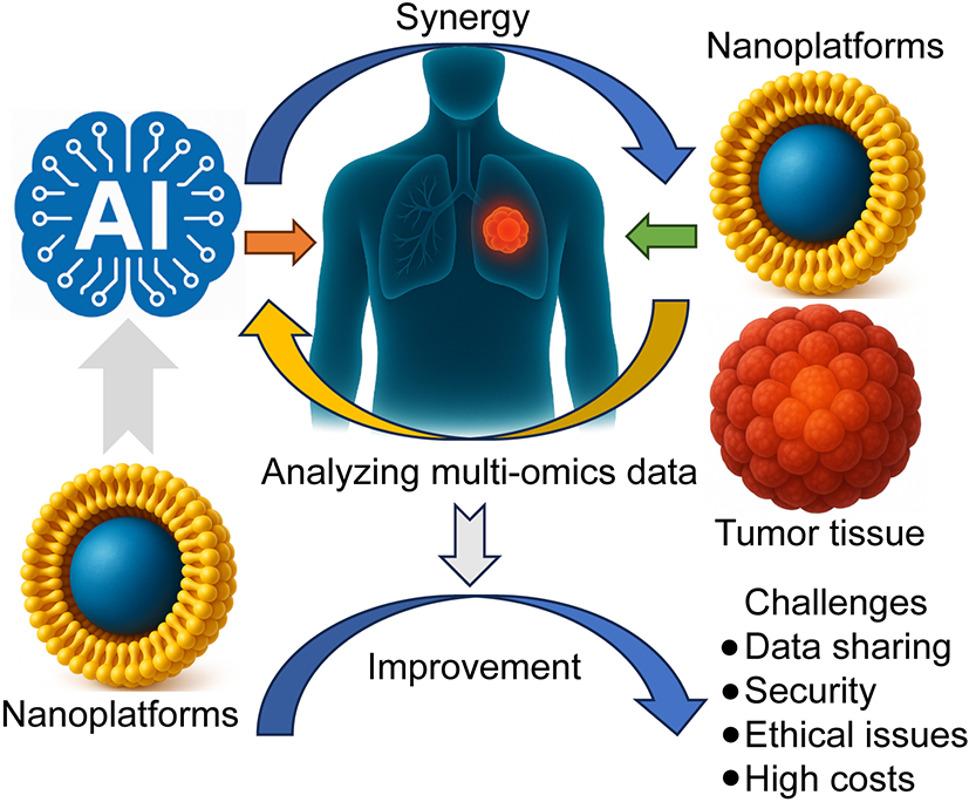

## Introduction

Cancer treatment remains challenging due to the biological complexity of the disease, limitations of current therapies, and marked interpatient variability [1]. A key obstacle is tumor heterogeneity: different cancer types harbor distinct molecular profiles, signaling pathways, and drug sensitivities, and even tumors of the same type can vary substantially between patients or among lesions within a single patient. These differences stem from genetic mutations, epigenetic alterations, and metabolic reprogramming—for example, EGFR or KRAS mutations and ALK fusions in lung cancer profoundly shape responses to targeted therapies [2, 3]. The tumor microenvironment adds another layer of complexity. This dynamic ecosystem of cancer cells, immune cells, fibroblasts, and stromal components critically modulates tumor behavior and treatment response [4]. Immunosuppressive features, such as elevated regulatory T cells or M2 macrophages, can blunt the efficacy of immune checkpoint inhibitors [5]. Current treatment modalities also have intrinsic limitations. Surgery mainly benefits early-stage solid tumors and is inadequate for invasive or metastatic disease. Chemotherapy lacks selectivity, damaging rapidly dividing normal cells and causing substantial toxicity, while resistance often undermines long-term benefit. Radiotherapy can provide effective local control, but off-target injury to surrounding tissues may lead to chronic complications, and some tumors—such as glioblastoma and pancreatic cancer—respond poorly. Collectively, the biological complexity of cancer and the shortcomings of existing therapies underscore the need for more precise, adaptable, and multidisciplinary strategies to improve clinical outcomes.

**Fig. 1:**
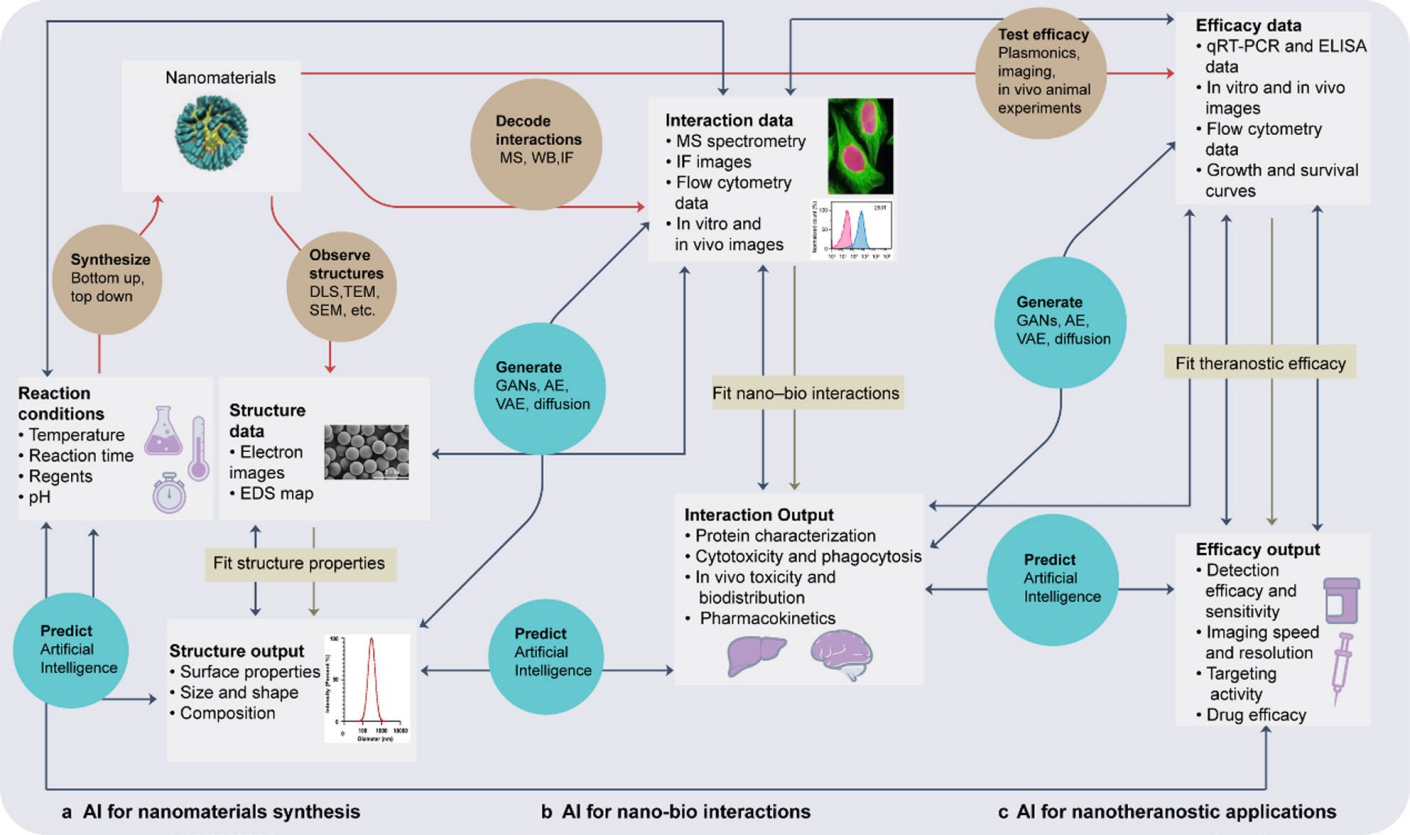
Workflow for AI-aided nanotheranostics [160–164]. (**a**) Nanomaterial synthesis using AI. (**b**) Understanding nano-bio interactions by AI. (**c**) Nanotheranostic applications. Red arrows represent the experimental process, brown arrows illustrate the fitting of experimental data, and dark blue arrows indicate both current applications of AI in prediction and generation, as well as future opportunities for AI in nanotheranostics. DLS, dynamic light scattering; TEM, transmission electron microscopy; SEM, scanning electron microscopy; EDS, energy dispersive spectroscopy; MS, mass spectrometry; WB, western blot; IF, immunofluorescence; qRT-PCR, quantitative reverse transcription PCR; ELISA, enzyme-linked immunosorbent assay; GANs, generative adversarial nets; AE, autoencoder; VAE, variational autoencoder

Nanotechnology and AI offer complementary strengths for tackling the complexity of cancer treatment. Nanotechnology advances precision medicine by enhancing tumor targeting, enabling controlled drug release, and integrating diagnosis with therapy [6]. Nanoparticles can sense tumor-associated cues—such as acidity or overexpressed receptors—to deliver drugs more selectively, reduce damage to healthy tissues, and maintain effective local drug levels through regulated on-site release [7]. Multifunctional nanosystems can further couple imaging and therapy, for example, magnetic nanoparticles that support magnetic resonance imaging (MRI)-based tumor localization while simultaneously carrying therapeutic agents. AI, in turn, integrates and analyzes large-scale genomic, transcriptomic, and imaging data. Deep learning improves early lesion detection and diagnostic accuracy [8, 9], while AI-driven virtual screening and molecular modeling help predict therapeutic response and toxicity, thus accelerating preclinical evaluation [10]. In the clinic, AI tools assist in dynamically tailoring treatment based on individual molecular profiles and response patterns, enhancing precision and personalization [11]. Together, AI and nanotechnology reinforce each other: AI guides rational nanomedicine design—such as material choice, particle size, and targeting ligands—whereas nanotechnology provides high-resolution biological and imaging data that further refine AI models [12]. This synergy supports more precise and adaptive strategies for cancer diagnosis and therapy (Fig. 1).

Cross-disciplinary integration is becoming increasingly important in cancer diagnosis and treatment, particularly in the combined application of AI and nanotechnology. AI supports diagnosis, treatment selection, and response evaluation through data analytics and predictive modeling, while nanotechnology enables advanced drug delivery, targeted therapy, and early detection. Together, they offer complementary strategies to overcome some limitations of conventional cancer therapies. For example, machine learning models can anticipate patient responses and guide treatment adjustment, whereas nanocarriers can bypass biological barriers and deliver drugs more precisely to tumors, improving efficacy and reducing adverse effects (Table 1). Despite this potential, the integration of AI and nanotechnology still faces major challenges, including technical complexity, ethical and regulatory issues, and limited clinical validation. This review summarizes the status of both fields in oncology, discusses key barriers to their combined use, and highlights future directions for cross-disciplinary collaboration to improve cancer care.

**Table 1:** Applications of AI-integrated nanomaterials in cancer diagnosis and treatment

Field of application	AI role	Nanomaterial Functions	Potential advantages	Refs
Diagnosis of tumors by imaging	Image recognition, pattern analysis, accurate segmentation	Nanoprobes, quantum dots, magnetic nanoparticles, gold nanoparticles, etc.	Improved imaging sensitivity and specificity for enhanced early diagnostics	[103, 173, 174]
liquid biopsy	Analyze circulating tumor DNA (ctDNA), exosomes, proteins, and other biomarkers	Nanosensors, nanoprobes, biofunctionalized nanoparticles, etc.	Enables non-invasive detection and improved detection sensitivity	[175–177]
Multimodal Image Fusion	AI fusion of multimodal data from MRI, CT, PET, optical imaging, etc.	Composite nanocontrast agents (fluorescent-magnetic, PET-CT bifunctional nanomaterials)	Improve image resolution and reduce false positives/negatives	[178–180]
Intelligent Drug Delivery	Predicting drug-nanocarrier interactions to optimize drug delivery strategies	Smart nanocarriers (liposomes, polymer nanoparticles, gold nanoparticles, etc.)	Improve drug targeting, reduce toxic side effects and increase treatment efficiency	[12, 181, 182]
Tumor Immunotherapy	Predicting immunotherapy response and optimizing immune cell activation strategies	Immune nano-adjuvants, nano-antigen presentation systems	Enhancement of tumor immune response to improve the effectiveness of individualized immunotherapy	[87, 103, 183]
Gene therapy	AI resolves gene editing targets to optimize siRNA/CRISPR designs	Gene delivery nanocarriers (lipid nanoparticles, polymer nanoparticles)	Improving the precision of gene therapy and reducing off-target effects	[184, 185]
Photothermal/Photodynamic Therapy	AI Calculation of optimal concentration of photosensitizer and irradiation conditions	Photothermal/photodynamic nanomaterials (noble metal nanoparticles, semiconductor quantum dots)	Increase treatment efficiency and reduce normal tissue damage	[186, 187]
Radiosensitization	Predicting radiotherapy tolerance and optimizing radiotherapy dose	Radiosensitizing nanomaterials (cerium oxide nanoparticles, gold nanoparticles)	Enhance radiation therapy effectiveness and reduce normal tissue damage	[188–190]
Individualized therapy	AI Builds Individualized Models to Predict Patient Response to Different Treatment Options	Smart nanoplatforms (nanomaterials that respond to changes in the physiological environment)	Improve individualized treatment outcomes and reduce ineffective treatment	[191–193]
Reversal of drug resistance	Analyzing drug resistance mechanisms to optimize combination therapy strategies	Drug resistance reversal nanocarriers (siRNA delivery, combination therapy nanoparticles)	Reducing tumor drug resistance and improving the efficacy of chemotherapy /targeted therapy	[194–196]
Intraoperative navigation	AI-assisted intraoperative imaging and analysis improves tumor resection accuracy	Intraoperative navigation of nanofluorescent probes, magnetic nanoparticles	Improvement of tumor resection precision and reduction of postoperative recurrence	[197–199]

## Status and development of nanotechnology in cancer therapy

### Core concepts and principles of nanotechnology

The use of nanotechnology in cancer therapy builds on the diversity of nanomaterials and their distinct physicochemical properties, mainly including organic, inorganic, and hybrid systems [13]. Organic nanomaterials such as polymers, lipids, and liposomes generally exhibit good biocompatibility and degradability, and can be surface-modified to improve targeting and reduce damage to normal tissues. Polymer-based nanoparticles allow tuning of drug loading and release by adjusting polymer chain length and molecular weight, while liposomes can encapsulate hydrophobic drugs to enhance stability and therapeutic selectivity [14]. Inorganic nanomaterials, including metal oxides, gold nanoparticles, and silica nanoparticles, are valued for their optical, magnetic, and electrical properties. Gold nanoparticles, for example, exploit surface plasmon resonance to act as drug carriers, imaging contrast agents and photothermal or photodynamic agents, enabling laser-triggered drug release and applications in hyperthermia, radiotherapy and photodynamic therapy [15, 16]. Hybrid nanomaterials combine features of both organic and inorganic components, allowing co-loading of anticancer drugs, adjustment of surface properties to enhance targeting, and use of magnetic or optical functions to improve treatment efficacy [17]. Through rational structural design and functional optimization, these three classes of nanomaterials can enhance targeting, solubility and therapeutic stability, providing a flexible toolkit for cancer treatment.

The therapeutic roles of nanomaterials in cancer depend not only on their composition and surface modifications but also on their underlying mechanisms of action. Among these, targeted delivery and the enhanced permeability and retention (EPR) effect are two core mechanisms of nanodrug delivery systems [18]. Targeted delivery involves decorating nanoparticle surfaces with specific ligands that recognize and bind receptors or antigens abnormally overexpressed on cancer cells, thereby enriching drugs at tumor sites, minimizing damage to normal tissues, and improving the efficacy of targeted chemotherapy and immunotherapy. The EPR effect arises from the abnormal vasculature, increased permeability, and poor lymphatic drainage of the tumor microenvironment, which facilitate passive extravasation and accumulation of nanoparticles in tumor regions—even for carriers without specific surface modifications [19]. The synergy between targeted delivery and the EPR effect greatly enhances the therapeutic potential of nanomedicines.

However, the EPR effect varies greatly across tumor types and individual patients, and its clinical relevance remains debated. Tumor heterogeneity, abnormal vasculature, dense extracellular matrix, and elevated interstitial fluid pressure all limit nanoparticle penetration and accumulation within tumors [20, 21], making passive targeting by unmodified nanoparticles insufficient for effective in vivo delivery. Consequently, research has increasingly shifted toward active targeting and microenvironment-responsive strategies—such as ligand modification, charge-reversible systems, and pH- or enzyme-sensitive carriers—combined with AI-driven optimization based on large-scale biological and physicochemical datasets. These approaches enable precise control over nanoparticle size, shape, and surface chemistry [22–24], helping to reduce the impact of EPR variability while improving the predictability and personalization of nanodrug delivery.

### The main application areas of nanotechnology

#### Nanotechnology in drug delivery: enhancing targeting and therapeutic efficiency

The application of nanotechnology in cancer treatment is primarily reflected in the innovation of drug delivery systems. Nanodrug carriers significantly enhance drug targeting, solubility, and bioavailability, while reducing side effects on normal tissues and improving therapeutic outcomes. Common nanocarriers include liposomes, polymer nanoparticles, and metal nanoparticles. Liposomes, composed of phospholipid bilayers, effectively encapsulate both water-soluble and hydrophobic drugs, preventing rapid drug clearance. Through surface modification, liposomes can specifically bind to receptors on the surface of cancer cells, enabling precise drug delivery. Polymer nanoparticles take advantage of the properties of polymer chains, offering excellent drug loading capacity and controlled release capabilities. By adjusting the molecular weight and hydrophilic-hydrophobic characteristics of the polymers, sustained release and targeted delivery can be achieved, enhancing anti-tumor efficacy. Metal nanoparticles (such as gold and silver nanoparticles) play an important role in cancer treatment due to their unique physicochemical properties (such as surface plasmon resonance effects) [25, 26]. They can not only serve as drug carriers but also locally heat the tumor area through photothermal effects, thereby killing cancer cells. The synergistic effect of liposomes, polymers, and metal nanoparticles in drug delivery has become one of the core technologies in precision cancer therapy, showing vast application potential.

#### Nanotechnology in cancer imaging and diagnosis: advancing precision visualization

In addition to drug delivery, nanotechnology also plays a significant role in cancer imaging and diagnosis [27]. Quantum dots and superparamagnetic nanoparticles are two types of nanomaterials commonly used in cancer imaging and diagnostics. Quantum dots are a class of semiconductor nanoparticles with unique optical properties, such as size-tunable fluorescence emission characteristics. Since quantum dots can be excited by light of different wavelengths and emit high-intensity light signals, they have broad applications in cellular imaging, in vivo imaging, and molecular imaging. By conjugating quantum dots with specific ligands, targeted imaging of tumor cells or specific molecules can be achieved, providing precise tumor localization and dynamic observation [28, 29]. Superparamagnetic nanoparticles, particularly iron oxide nanoparticles, are highly responsive to magnetic fields and are often used in MRI [30]. As MRI contrast agents, superparamagnetic nanoparticles can significantly enhance image contrast, enabling clearer visualization of tumor structures and localization. Additionally, superparamagnetic nanoparticles are widely applied in photoacoustic imaging, where their excellent performance in the photoacoustic effect allows non-invasive imaging of deep tumor tissues. These nanoparticles provide important technical support for the early diagnosis of cancer, pathological classification, and the assessment of therapeutic efficacy during treatment, making them highly valuable for clinical applications.

#### Nanotechnology in cancer immunotherapy: enhancing immune activation and precision modulation

Nanotechnology has also shown tremendous potential in the field of cancer immunotherapy. Immunotherapy is a treatment approach that attacks cancer cells by modulating the patient’s immune system and has become an important direction in cancer treatment in recent years. The application of nanocarriers can effectively enhance immune responses and improve the efficacy of immunotherapy. By utilizing nanocarriers to deliver antigens or immune modulatory molecules, the immune system’s ability to recognize and attack tumors can be increased. For example, using nanoparticles to deliver tumor-specific antigens can activate the patient’s immune system, enabling it to recognize and attack cancer cells [31].

Additionally, nanocarriers can be used to deliver immune-modulatory molecules, such as cytokines or immune checkpoint inhibitors, to modulate the tumor microenvironment and enhance the anti-tumor capabilities of immune cells [32, 33]. Through the precise control of immunotherapy via nanocarriers, not only can treatment efficacy be increased, but the side effects of immunotherapy can also be reduced. Recent studies have shown that nano-immunotherapy has achieved significant results in various cancer models, particularly when combined with other therapeutic methods (such as radiotherapy or chemotherapy), where it can synergistically enhance the therapeutic effects on tumors [34, 35]. This makes the application of nanotechnology in cancer immunotherapy highly promising, positioning it as an important component of personalized treatment and precision medicine.

### Key technology bottlenecks and unmet needs

Although significant progress has been made in the development of nanotechnology for cancer treatment, several technical bottlenecks and unmet needs remain. First, biocompatibility and toxicity issues are among the core challenges in the clinical translation of nanomaterials. Many nanomaterials—particularly inorganic nanoparticles and certain hybrid formulations—can induce inflammatory and immune responses or cause accumulation-related toxicity within the body. In particular, metal-based nanoparticles that persist in tissues for extended periods may result in hepatic and renal impairment, as well as dysfunction of other organs [36]. Even materials considered to have good biocompatibility, such as liposomes and polymer nanoparticles, may exhibit potential toxicity under certain conditions due to their metabolic byproducts. Therefore, improving the biocompatibility of nanomaterials and reducing their toxicity is one of the key areas of current research. On the one hand, optimizing the surface chemical properties and physical morphology of nanomaterials, such as surface coating with hydrophilic polymers (e.g., polyethylene glycol) or using natural materials for synthesis, can significantly enhance their stability and safety *in vivo* [37]. On the other hand, conducting comprehensive studies on the metabolic pathways and long-term biological impacts of nanomaterials will help better assess their potential risks, promoting the safe application of nanotechnology in cancer therapy [13].

The demand for improved targeted delivery efficiency is another significant bottleneck faced by nanotechnology in cancer treatment. Although targeted delivery technologies, through specific ligand modifications or EPR effects, have significantly increased drug accumulation in tumor tissues, their actual efficiency in the complex in vivo environment often falls short of expectations. Many nanoparticles are affected by protein adsorption in the bloodstream, forming a “protein corona,” which may lead to their phagocytosis by macrophages or capture by other non-target tissues, preventing them from reaching the tumor target area [38]. Additionally, the high interstitial pressure and heterogeneity within tumor tissues limit the uniform distribution and deep penetration of nanodrugs. To enhance targeted delivery efficiency, current research is focused on developing novel smart nanomaterials, such as nanoparticles that are sensitive to external stimuli (e.g., pH, temperature, light, electromagnetic fields), which can release drugs in specific tumor environments [39]. Meanwhile, surface functionalization strategies for nanomaterials are also continuously optimized. By precisely designing ligands or antibody binding, the binding efficiency to targeted receptors can be improved [40]. These technological advancements hold the potential to overcome the limitations of current delivery technologies and further enhance the therapeutic efficacy of nanodrugs.

The intervention and functional optimization of the complex tumor microenvironment is another important challenge faced by nanotechnology in cancer treatment. The heterogeneity and dynamic changes of the tumor microenvironment, such as hypoxia, high lactate levels, abnormal vascular structures, and immunosuppressive environments, significantly affect the efficacy of nanomaterials at the tumor site. For example, hypoxic conditions may weaken the effectiveness of photodynamic therapy and radiotherapy, while the immunosuppressive environment reduces the effectiveness of tumor immunotherapy [41]. To address this challenge, researchers have been actively developing multifunctional nanomaterials capable of modulating the tumor microenvironment in a synergistic manner. For example, oxygen-carrying nanoparticles can deliver and gradually release oxygen within hypoxic tumor regions, thereby alleviating hypoxia and enhancing therapeutic efficacy [42]. Nanoparticles with immune-regulating functions can deliver cytokines or immune checkpoint inhibitors to activate antitumor immune responses [43]. Furthermore, nanomaterials that respond to tumor microenvironment-specific stimuli (such as acidity, reductive conditions, or enzyme concentrations) can precisely release drugs or activate therapeutic functions at the tumor site [44]. These tumor microenvironment-based optimization strategies offer new approaches to overcoming the limitations of traditional treatments and hold the potential to significantly enhance the overall efficacy of cancer therapy.

## Status of artificial intelligence in cancer research

### Core AI capabilities

#### AI-driven integration of multi-omics data

One of the core capabilities of AI in cancer treatment is its efficient processing and integrative analysis of multi-omics data. The occurrence and progression of cancer is a complex biological process driven by multiple factors, involving data from various layers such as genomics, transcriptomics, proteomics, and metabolomics [45, 46]. These multi-omics data are large in volume and highly heterogeneous, making it difficult for traditional analysis methods to fully uncover the underlying patterns. AI technologies, particularly machine learning and deep learning algorithms, can extract complex associative patterns from large-scale data, integrate data from different omics layers, and construct precise disease models. For example, by integrating genetic mutations, transcription factor expression levels, and metabolic pathway changes in cancer patients, AI can identify molecular features associated with specific cancer subtypes, aiding clinical efforts in more precise patient stratification and risk prediction [47, 48]. Furthermore, AI technologies can continuously update analysis models to accommodate newly available data, enhancing the predictive capability of the models [49]. This approach of multi-omics data integration not only enhances the understanding of cancer’s biological mechanisms but also provides data support for personalized treatment, driving the development of precision medicine.

#### AI-enhanced cancer imaging

AI’s application in cancer image analysis has significantly improved the accuracy of early diagnosis and disease staging. Based on deep learning algorithms, AI can automate the analysis of cancer imaging data (such as CT, MRI, PET-CT, etc.), enabling efficient detection, feature extraction, and classification of lesions [50]. Compared to traditional manual image review, AI technology demonstrates higher efficiency and consistency when processing large-scale image data [51]. For example, in lung cancer screening, AI algorithms can rapidly identify the morphological features of pulmonary nodules and assess their malignancy potential, reducing the incidence of missed and misdiagnosed cases [52]. Furthermore, AI can accurately stage cancer and predict its metastatic risk by analyzing imaging features, thereby providing a basis for developing personalized treatment plans. In recent years, some AI tools have integrated radiomics technology, extracting vast amounts of high-dimensional data from images, which further enhances the early diagnostic capabilities for cancer [53, 54]. These deep learning-based image analysis technologies not only alleviate the workload of clinicians but also significantly improve the accuracy of cancer diagnosis and staging, driving the standardization and personalization of cancer treatment.

#### AI-driven drug discovery and molecular design

In the field of drug design, AI has become a core tool for virtual screening and molecular property prediction [55–57]. Traditional drug development processes are time-consuming and costly, whereas AI significantly shortens the drug discovery timeline by accelerating virtual drug screening. By learning interaction patterns between known drug molecules and their targets, AI models can predict the binding affinity of new molecules to specific targets, enabling rapid identification of potential drug candidates. For instance, algorithms such as generative adversarial networks (GANs) and recurrent neural networks (RNNs) can generate compound structures with high activity and low toxicity based on the characteristics of target molecules [58, 59]. Additionally, AI can predict the physicochemical properties, biological activity, and pharmacokinetic characteristics of molecules, thereby optimizing their clinical performance [60]. In recent years, AI has demonstrated immense potential in cancer drug discovery, including the identification of novel small-molecule drug targets and the development of precise immunotherapies. These advancements not only enhance drug development efficiency but also significantly reduce costs, providing cancer patients with more innovative treatment options. AI-driven drug design technologies are injecting new momentum into global cancer research.

### Applications of AI in cancer

#### Applications in cancer diagnosis

Deep learning models have achieved significant breakthroughs in early cancer screening, establishing themselves as a critical advancement in cancer diagnosis. In lung cancer screening, deep learning-based algorithms efficiently analyze low-dose spiral CT (LDCT) images, automatically detecting pulmonary nodules and assessing their malignancy risk. For example, a deep learning model developed by Google has demonstrated diagnostic accuracy comparable to that of radiologists by analyzing large-scale pulmonary imaging data, while significantly reducing missed diagnoses [61]. Additionally, this technology can track dynamic changes in pulmonary nodules, providing long-term follow-up recommendations for patients. In the field of breast cancer, deep learning algorithms for automated mammography analysis have been shown in multiple studies to improve early cancer detection rates while reducing unnecessary biopsies [51]. For skin cancer detection, deep learning-based image recognition models can rapidly analyze skin lesion images, achieving diagnostic accuracy comparable to that of dermatologists, particularly in distinguishing malignant melanoma from benign skin conditions [62]. The successful application of these deep learning models in lung, breast, and skin cancer has not only significantly enhanced the efficiency and precision of early screening but has also provided technological support for improving patient survival rates.

AI has demonstrated significant potential in aiding the discovery and clinical validation of cancer biomarkers. Biomarkers serve as crucial tools for cancer diagnosis and treatment; however, their traditional discovery process is often time-consuming and resource-intensive. AI can efficiently integrate multi-omics data to rapidly identify potential biomarkers closely associated with cancer development and progression. For instance, AI models can detect novel mutation sites from cancer patients’ genomic data, screen specific gene expression patterns from transcriptomic data, or identify characteristic metabolites from metabolomic data [63]. Once experimentally validated, these biomarkers can be used for early cancer diagnosis or prognostic assessment. In recent years, AI has also been utilized to predict the correlation between biomarkers and patient treatment responses, thereby guiding the development of personalized therapeutic strategies. For example, AI models analyzing gene expression patterns associated with immune checkpoint inhibitor efficacy have successfully identified biomarkers closely linked to immunotherapy response [64, 65]. Furthermore, AI-assisted biomarker discovery has accelerated the advancement of liquid biopsy techniques. By integrating circulating tumor DNA (ctDNA), exosomes, and protein biomarkers from blood samples, AI enhances the precision of non-invasive cancer diagnostics [66, 67]. These technological advancements have created new opportunities for clinical cancer diagnosis and treatment, driving the rapid development of precision medicine.

#### Potential contribution in treatment decisions

AI has demonstrated remarkable potential in generating precision treatment strategies, particularly in drug combination recommendations. Cancer treatment often requires multiple drugs to be used in combination to maximize tumor suppression while minimizing drug resistance. However, traditional drug combination design relies heavily on clinical trial experience and single-target research, making the process time-consuming and costly. By analyzing large-scale clinical and omics data, AI can rapidly predict drug interactions and identify optimal combination therapies. For example, AI models can recommend personalized drug combinations based on a patient’s genetic mutation profile, tumor microenvironment characteristics, and prior treatment history, significantly enhancing treatment precision [9, 68]. Additionally, AI can simulate the efficacy and side effects of different drug combinations, assisting physicians in selecting the most suitable treatment plan. In recent years, AI systems based on deep learning and reinforcement learning have been applied to drug combination recommendations. For instance, IBM Watson for Oncology can provide personalized treatment suggestions by integrating patients’ molecular profiles with international guidelines [69]. A recent collaborative study by Macau University of Science and Technology and Sun Yat-sen University proposed a multimodal AI foundation model based on a Transformer-CNN hybrid architecture. By integrating pathological and clinical data, the model can accurately predict pathological complete response and disease-free survival in breast cancer patients receiving neoadjuvant chemotherapy, providing intelligent support for personalized treatment decisions [70] The application of these AI-driven technologies not only optimizes the decision-making process in cancer treatment but also provides physicians with scientific evidence, offering the potential to significantly improve patient outcomes.

AI also plays a crucial role in assisting with efficacy assessment and side effect monitoring. Cancer treatment outcomes and adverse effects vary significantly among individuals, making real-time monitoring and dynamic evaluation essential for optimizing therapeutic strategies. By integrating patients’ imaging data, laboratory indicators, and self-reported symptoms, AI can efficiently assess treatment progress and effectiveness. For example, by analyzing follow-up imaging data of cancer patients, AI can automatically quantify tumor size changes and predict treatment response trends [71, 72]. Additionally, AI can rapidly detect potential drug resistance by analyzing patients’ gene expression profiles or circulating tumor DNA (ctDNA) levels in the blood, providing timely information for treatment adjustments. In terms of side effect monitoring, AI leverages natural language processing to analyze patients’ electronic health records or online feedback, automatically identifying potential adverse drug reactions and aiding physicians in formulating appropriate interventions. For instance, some AI systems can detect and predict chemotherapy-related toxicities, such as bone marrow suppression or neurotoxicity, in real-time, guiding dose adjustments or drug substitutions [73]. These AI-driven efficacy assessment and side effect monitoring technologies not only enhance treatment safety and effectiveness but also significantly reduce patient discomfort, laying the foundation for personalized and dynamically optimized cancer therapy.

### Current limitations and technical barriers

Data standardization and sharing represent one of the key bottlenecks limiting the application of AI in cancer diagnosis and treatment [74]. AI models rely on large-scale, high-quality datasets, yet cancer-related data are highly heterogeneous—spanning imaging, multi-omics, and clinical follow-up information. Differences in data formats, naming conventions, acquisition procedures, and quality-control standards across institutions severely hinder data integration and interoperability [75]. In addition, privacy and security requirements mean that data sharing must undergo strict ethical review and de-identification processes, increasing cost and time while potentially compromising data integrity and usability [76].

To address these challenges, TCGA has established comprehensive genomic and clinical databases across multiple tumor types, advancing machine-learning-based tumor classification and biomarker discovery [77]. The GDC integrates multi-omics data through a unified metadata structure, improving reproducibility and comparability, while the ICGC promotes global collaborative research through multi-institutional coordination and open data access. These initiatives provide important models for data standardization, interoperability, and transparency, and they lay a solid foundation for intelligent healthcare systems as well as the design, optimization, and evaluation of multifunctional nanoplatforms. However, achieving high-quality, cross-institutional and cross-border data sharing on a global scale will still require continued progress in technical standards, legal and ethical frameworks, and regulatory mechanisms.

AI faces two major barriers in its clinical translation within oncology and nanomedicine: the " black-box " of models and safety concerns [78, 79]. Deep learning models often lack transparent decision-making processes and cannot be easily aligned with established medical knowledge, which reduces clinicians’ trust in AI-generated findings for imaging, pathology, and treatment decisions. In addition, model errors caused by biased training data or noisy inputs can pose significant risks in cancer treatment. Therefore, it is essential to adopt explainable AI (XAI) approaches—such as visualizing attention regions and displaying feature importance—along with multi-level validation and regulatory mechanisms, to enhance model robustness, safety, and clinical acceptability.

At the application level, platforms such as IBM Watson for Oncology, Google DeepMind Health, and PathAI have already been used for clinical decision support, medical image analysis, and pathological diagnosis assistance, while frameworks like TensorFlow and PyTorch support multimodal cancer data modeling [80]. However, broader deployment of these technologies—and their extension into nanomedicine—remains limited by cross-institutional data heterogeneity, lack of unified standards, and privacy and regulatory constraints. Approaches such as federated learning, secure multiparty computation, and XAI are increasingly employed to enable multi-center collaborative modeling without exposing raw data, while model governance and visualization techniques enhance system robustness, fairness, and trustworthiness. Together, these developments lay the methodological foundation for deeper integration of AI with multifunctional nanoplatforms.

## Cross-disciplinary integration of AI and nanotechnology in cancer treatment

### AI-enabled nanotechnology design and optimization

**Fig. 2:**
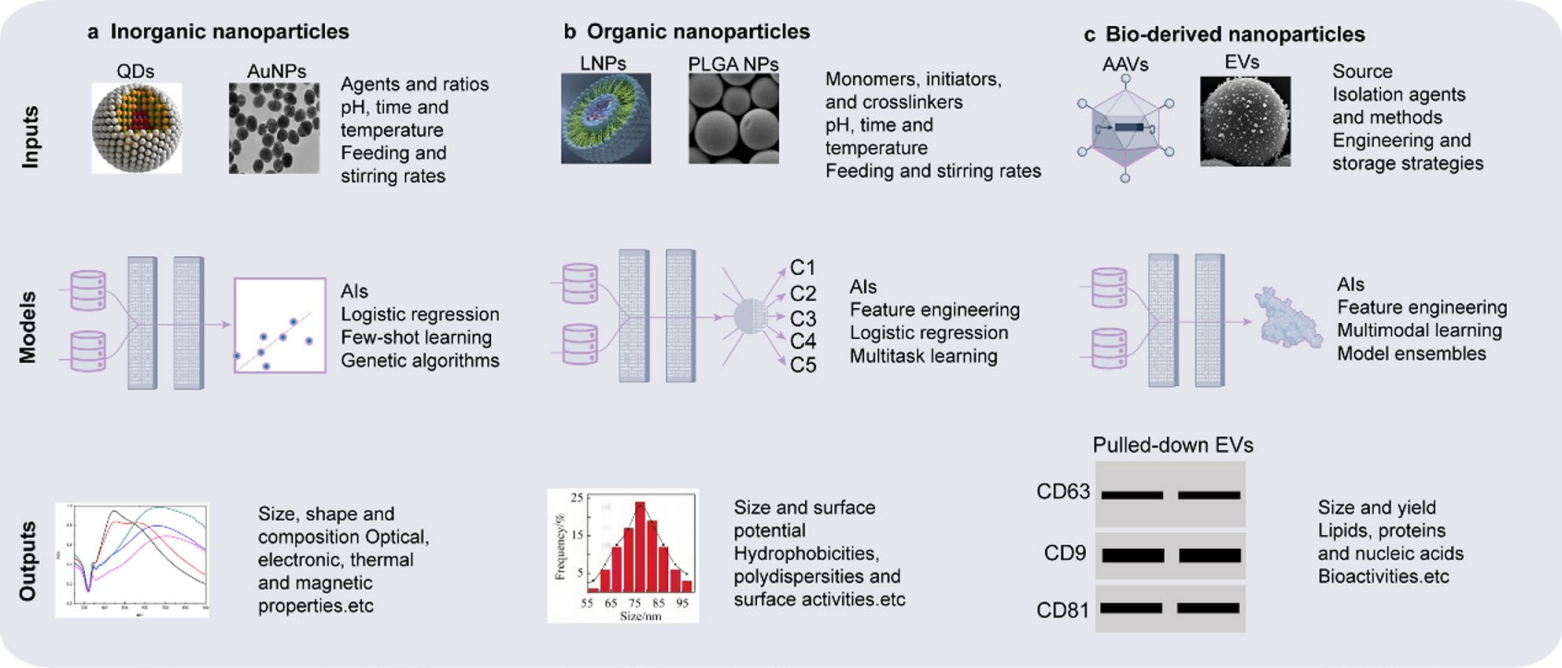
AI-assisted synthesis of different types of nanoparticles [160, 161, 165, 166]. Examples of AI model inputs, architectures, and outputs related to inorganic (**a**), organic (**b**), and biological (**c**) nanoparticle synthesis. C1–C5 indicate class 1–class 5. QDs, quantum dots; LNPs, lipid nanoparticles; PLGA NPs, Poly lactic-co-glycolic acid nanoparticles; AAVs, Adeno-Associated Viruses; EVs, extracellular vesicles

**Fig. 3:**
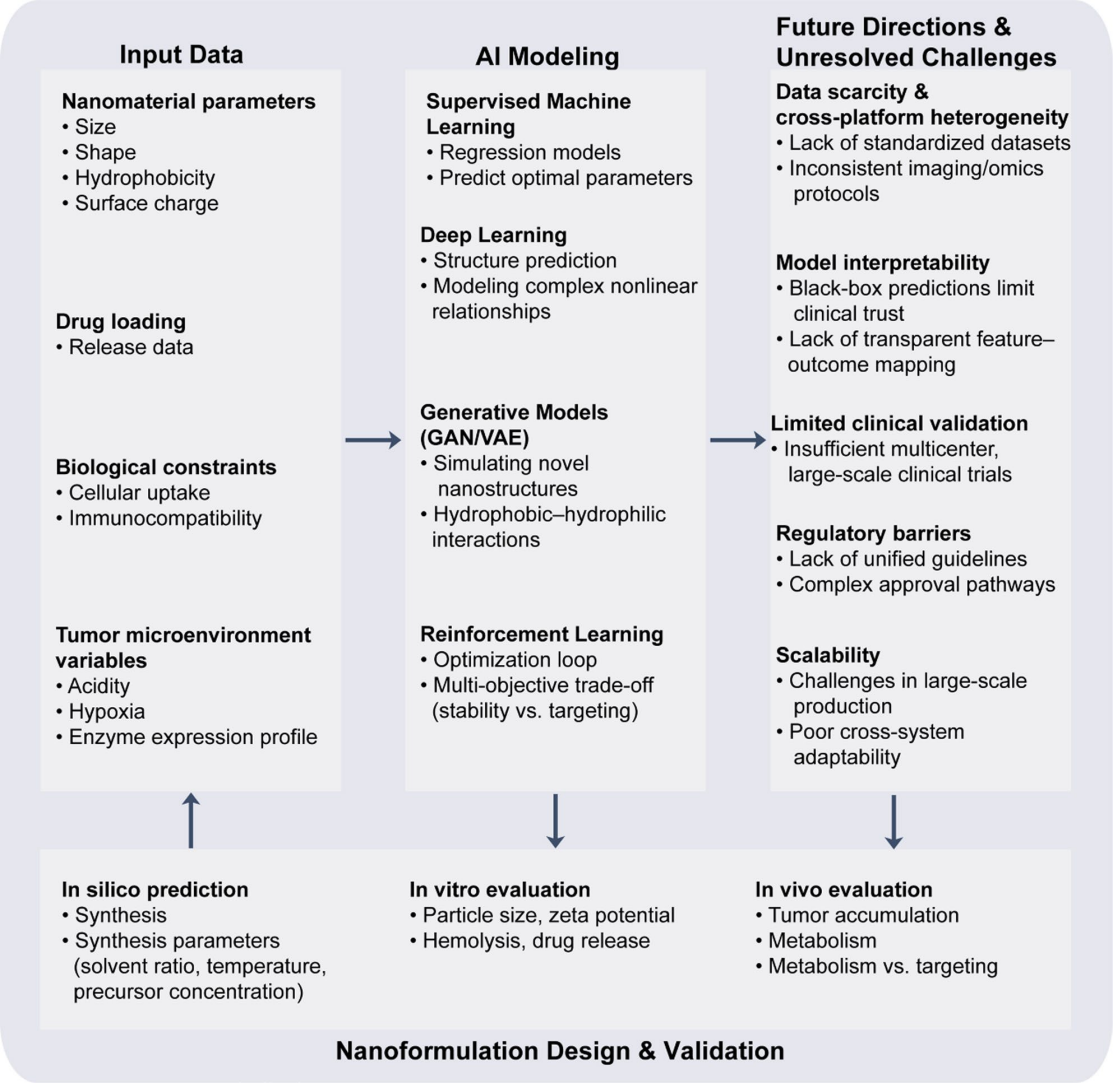
AI-driven optimization workflow for nanoformulation design [22, 24, 105]. AI models integrate nanomaterial parameters, drug loading profiles, biological constraints and tumor microenvironment variables to predict optimal nanoparticle designs. The resulting formulations are iteratively refined through in silico prediction and in vitro/in vivo evaluation, while data scarcity, model interpretability, regulatory and scalability issues remain key challenges

AI is becoming a central tool for the rational design of nanomaterials, enabling systematic optimization of nanoparticle physicochemical properties across material screening, structural prediction, and process engineering to improve tumor targeting, drug-loading efficiency, and reduce nonspecific distribution [81, 82]. Supervised learning models can map large-scale synthesis parameters (Fig. 2)—such as precursor concentration, solvent ratio, temperature, and pH—to key nanoparticle features including size, ζ-potential, morphology, and encapsulation efficiency. Algorithms such as random forests, SVR, and XGBoost are used to identify optimal conditions, producing high-performance nanocarriers with sizes consistently within 50–150 nm and surface charges between − 10 and + 10 mV, balancing the EPR effect, in vivo stability, and uniform biodistribution [83–85]. In addition, AI can predict nanoparticle solubility and stability, guiding formulation development for complex in vivo environments [86]. These AI-driven strategies significantly shorten the design cycle and enhance overall material performance, providing a strong foundation for the application of nanotechnology in cancer therapy (Fig. 3).

At the molecular recognition level, deep learning-driven de novo protein design has further enhanced the precision of tumor-associated target identification. To address issues such as cross-reactivity and batch variability observed with traditional antibodies in extracellular vesicle (EV) detection, researchers at Harvard Medical School developed AI-designed nanobinders (DNBs) capable of highly specific recognition of EV target proteins. For example, in detecting PD-L1, DNBs increased imaging signal intensity by approximately 51-fold compared with conventional methods, significantly improved detection sensitivity, and markedly reduced nonspecific binding. Moreover, DNBs exhibited potential for immune checkpoint blockade, highlighting their promise as antibody alternatives for EV analysis and cancer diagnosis and therapy [87].

AI can also predict the in vivo distribution and drug-release behavior of nanomaterials, thereby optimizing their therapeutic performance. The fate of nanomedicines is shaped by the tumor microenvironment, biological barriers (e.g., hepatic and renal clearance), and material properties [20, 88–91]. Conventional biodistribution studies rely on animal experiments, which are costly and offer limited extrapolability. By integrating large-scale experimental data with biological simulation models, AI can more precisely forecast nanoparticle biodistribution. For instance, graph neural network–based algorithms can learn the relationship between nanoparticle structure and biodistribution to predict tumor accumulation [92]. AI can also simulate drug release under different administration routes, helping to optimize release rate and timing. Deep learning models further enable the design of stimuli-responsive nanocarriers that release drugs efficiently in response to tumor-specific cues such as acidity or enzyme levels [89, 93–96].These capabilities make AI a powerful tool for nanomedicine design and optimization, helping to address key challenges in tumor therapy—particularly targeted delivery and controlled drug release—and are expected to accelerate the clinical translation of nanotechnology in cancer treatment.

### AI-driven intelligent response nano systems

AI applications in the design of stimuli-responsive nanomaterials open new avenues for optimizing material performance, especially by simulating the dynamic tumor microenvironment. Factors such as low pH, local temperature gradients, and overexpressed enzymes place stringent demands on nanomaterial responsiveness, whereas traditional, largely static design strategies struggle to capture this complexity [97]. Machine learning models can learn patterns of microenvironmental change from experimental data and guide the creation of materials that adapt dynamically. For example, AI can help design pH-sensitive polymeric nanoparticles that rapidly reconfigure and release drugs in acidic tumor regions [98], or enzyme-responsive systems that initiate drug delivery upon encountering specific enzymatic activity [94]. It can also assist in optimizing thermosensitive formulations—such as heat-triggered liposomes that release drugs in response to tumor-associated temperature changes [99]. By leveraging AI-driven simulation and prediction, the design efficiency of these stimuli-responsive nanomaterials is greatly improved, providing a stronger foundation for precise targeting and controlled drug release in cancer therapy.

AI-driven real-time feedback has greatly strengthened the regulatory capacity of intelligent responsive nanosystems in tumor therapy. A defining feature of these systems is their ability to dynamically adjust function during treatment based on real-time signals. In photodynamic therapy (PDT), for example, they can modulate treatment intensity according to local light dose, oxygen levels, or temperature at the tumor site [100]. Traditional feedback often depends on offline analysis, limiting real-time control. By integrating sensor outputs with deep learning algorithms, AI enables on-the-fly analysis of multidimensional treatment data and rapid generation of adjustment strategies. In PDT, AI can continuously monitor photosensitizer activation and intratumoral oxygen levels, predict therapeutic efficacy, and adapt laser dosage accordingly, thereby improving tumor eradication while reducing injury to healthy tissue [101]. Similarly, AI can analyze real-time biological signals (such as temperature or pH) to dynamically control the release rate of thermo- or chemotherapeutic agents, enhancing both safety and precision [102]. These AI-based feedback mechanisms not only optimize therapeutic outcomes but also provide a new technical pathway for personalized treatment, underscoring the clinical potential of intelligent responsive nanosystems.

In recent years, multiple studies have demonstrated the significant translational potential of AI-driven intelligent, responsive nanosystems in cancer therapy. Using the deep learning platform Gramord, researchers at the Shanghai Institute of Materia Medica (CAS) virtually screened ~1.6 million molecular pairs from ~1,800 natural small molecules and identified oridonin (Ori) and cepharanthine (Cep) as an optimal self-assembling combination, forming excipient-free nanoparticles (OCN) capable of direct tumor killing, inducing immunogenic cell death (ICD), and activating the STING pathway to generate durable immune memory—breaking the traditional trial-and-error paradigm of natural-product nanomedicine development [103]. At the Indian Institute of Technology (BHU) Varanasi, machine-learning interatomic potentials (MLIP) from the ORB suite were used to replace costly DFT calculations, efficiently identifying stable and energetically favorable Dox/Co-Al LDH nanostructures that achieved ~80% cytotoxicity against melanoma cells with only ~8% toxicity to normal cells, enabling highly selective therapy [104]. Furthermore, ML–DFT integrated screening identified RuO₂ nanoparticles from a large nanozyme library as a non-inflammatory photothermal agent; under NIR-II irradiation, RuO₂ NPs combine high photothermal conversion with CAT-like activity to clear ROS, downregulate inflammatory factors, and maintain excellent biocompatibility [105]. Collectively, these examples illustrate how the convergence of AI and responsive nanotechnology accelerates the path from conceptual design to clinical translation, laying a solid foundation for intelligent and adaptive cancer therapy.

### Collaboration between AI and nanotechnology in tumor microenvironment regulation

Nanotechnology has demonstrated a multifaceted and significant role in the regulation of the tumor microenvironment (TME), offering new breakthroughs for cancer treatment [106]. The TME is a key regulatory factor in tumor growth and progression, comprising a complex composition of immune cells, blood vessels, extracellular matrix, and redox balance [107]. Nanotechnology intervenes in these microenvironmental characteristics effectively by developing nanomaterials with specific functions. In tumor immunity regulation, nanoparticles can serve as delivery platforms to precisely transport antigens or immunomodulatory molecules to tumor sites, thereby activating anti-tumor immune responses [108]. Additionally, immune checkpoint inhibitors (such as PD-1/PD-L1 inhibitors) delivered via nanotechnology significantly enhance drug efficacy [109]. In angiogenesis, abnormal tumor vasculature often affects drug delivery efficiency. Nanoparticles can regulate the activity of angiogenic factors (such as VEGF) through specific formulations, improving vascular structure and enhancing drug delivery efficiency [110, 111]. Nanotechnology also plays a role in regulating the tumor’s redox balance. By designing responsive nanocarriers, it can precisely deliver reactive oxygen species (ROS) or reductive molecules to disrupt the tumor cells’ antioxidant mechanisms, further promoting tumor cell apoptosis [112]. The integration of these technologies not only effectively inhibits tumor progression within the local microenvironment but also provides new possibilities for overcoming the limitations of traditional therapies.

AI, through modeling, predicts key molecular changes within the tumor microenvironment (TME), providing strong support for optimizing treatment strategies using nanotechnology [113]. The dynamic and heterogeneous nature of the TME presents significant challenges in developing treatment strategies, but AI technology, by integrating multi-omics data and dynamic monitoring information, can accurately predict the trends of key molecular changes in the microenvironment. AI models can simulate immune cell infiltration within the TME, predict the impact of various treatment regimens on the immune microenvironment, and guide the optimization of immuno-nanotherapies [114]. For angiogenesis, AI can model and analyze the hemodynamic characteristics of the tumor site, predict the distribution of nanoparticle carriers, and assess drug release efficiency, thereby optimizing treatment plans [84]. Additionally, AI can analyze redox status data from the tumor site, predict changes in ROS levels and antioxidant enzyme activity, and provide a basis for designing efficient oxidative stress-based nanotherapies [45]. This collaboration between AI and nanotechnology not only accelerates the development of treatment strategies but also enables personalized and precise treatments by dynamically adjusting therapeutic parameters. This bidirectional integration model offers a new technological pathway for overcoming the complex therapeutic challenges associated with the tumor microenvironment and demonstrates the immense potential of future cancer therapies.

### AI optimized diagnosis and treatment integrated nanoplatform

**Fig. 4:**
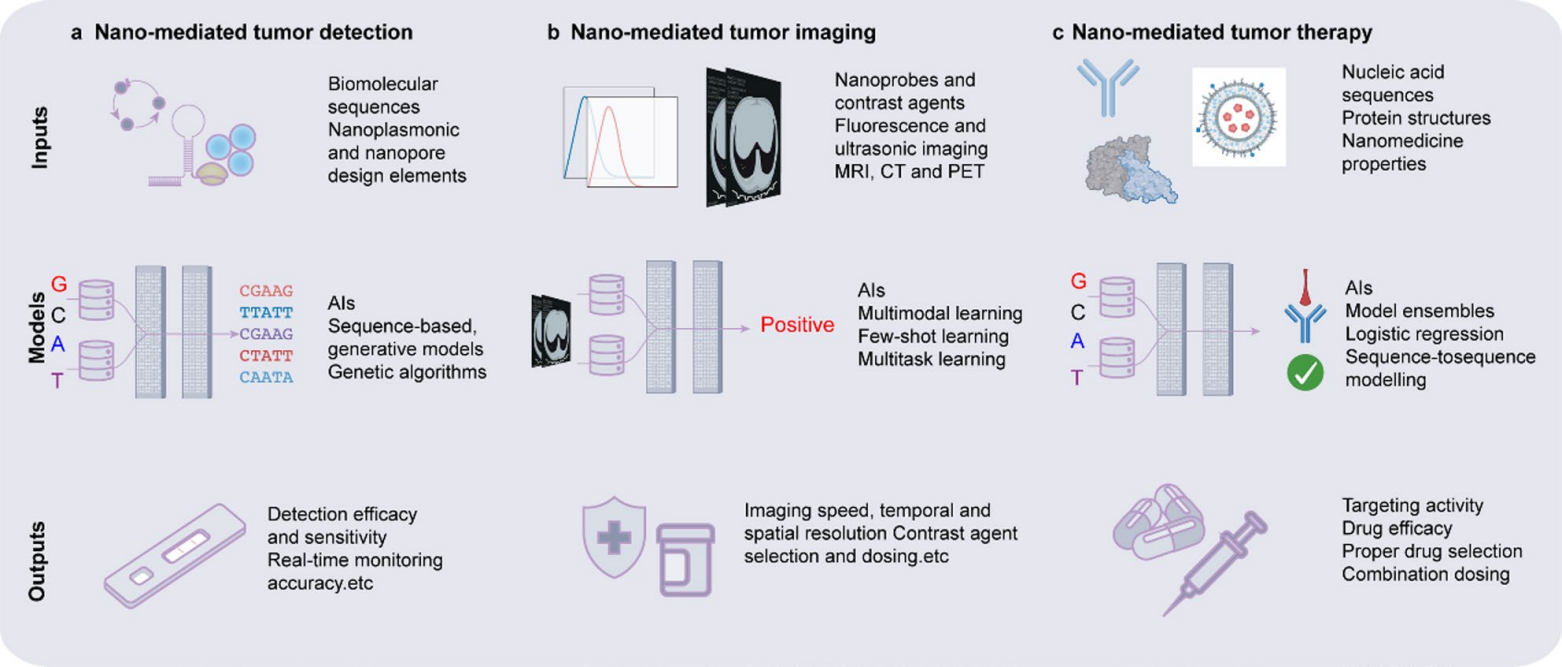
AI-assisted nanotheranostic in tumor [50, 167–169]. Examples of AI model inputs, architectures, and outputs related to nano-mediated tumor detection (**a**), imaging (**b**), and therapy (**c**). MRI, magnetic resonance imaging; CT, computed tomography; PET, positron emission tomography

The AI-optimized integrated diagnostic and therapeutic nanoplatform combines nanoparticle imaging agents and therapeutic molecules, realizing a new model for precise diagnosis and treatment [115, 116]. Traditional cancer diagnosis and treatment are typically separate processes, but the integrated diagnostic and therapeutic platform seamlessly connect these two stages using nanotechnology, enabling more efficient treatment. The combination of nanoparticle imaging agents and drug carriers allows for precise tumor imaging and targeted therapy simultaneously [117–119]. Materials such as magnetic nanoparticles and quantum dots, known for their excellent imaging properties, are widely used in MRI and fluorescence imaging [120]. When these imaging agents are combined with chemotherapy drugs or photodynamic therapy molecules, they not only enable real-time tracking of tumor locations but also monitor drug release and therapeutic efficacy in real-time. AI plays a central role in this process, dynamically adjusting treatment parameters such as drug dosage, photodynamic activation timing, or temperature control through real-time analysis of imaging data. This AI-based real-time feedback mechanism significantly enhances treatment precision and safety, preventing overtreatment or undertreatment. Additionally, AI can predict the potential efficacy or side effects of treatment based on the patient’s specific imaging features, providing a reliable basis for personalized therapy. The integration of AI in the diagnostic and therapeutic nanoplatform not only improves treatment efficiency but also reduces side effects on healthy tissue, opening new pathways for comprehensive cancer therapy (Fig. 4).

**Fig. 5:**
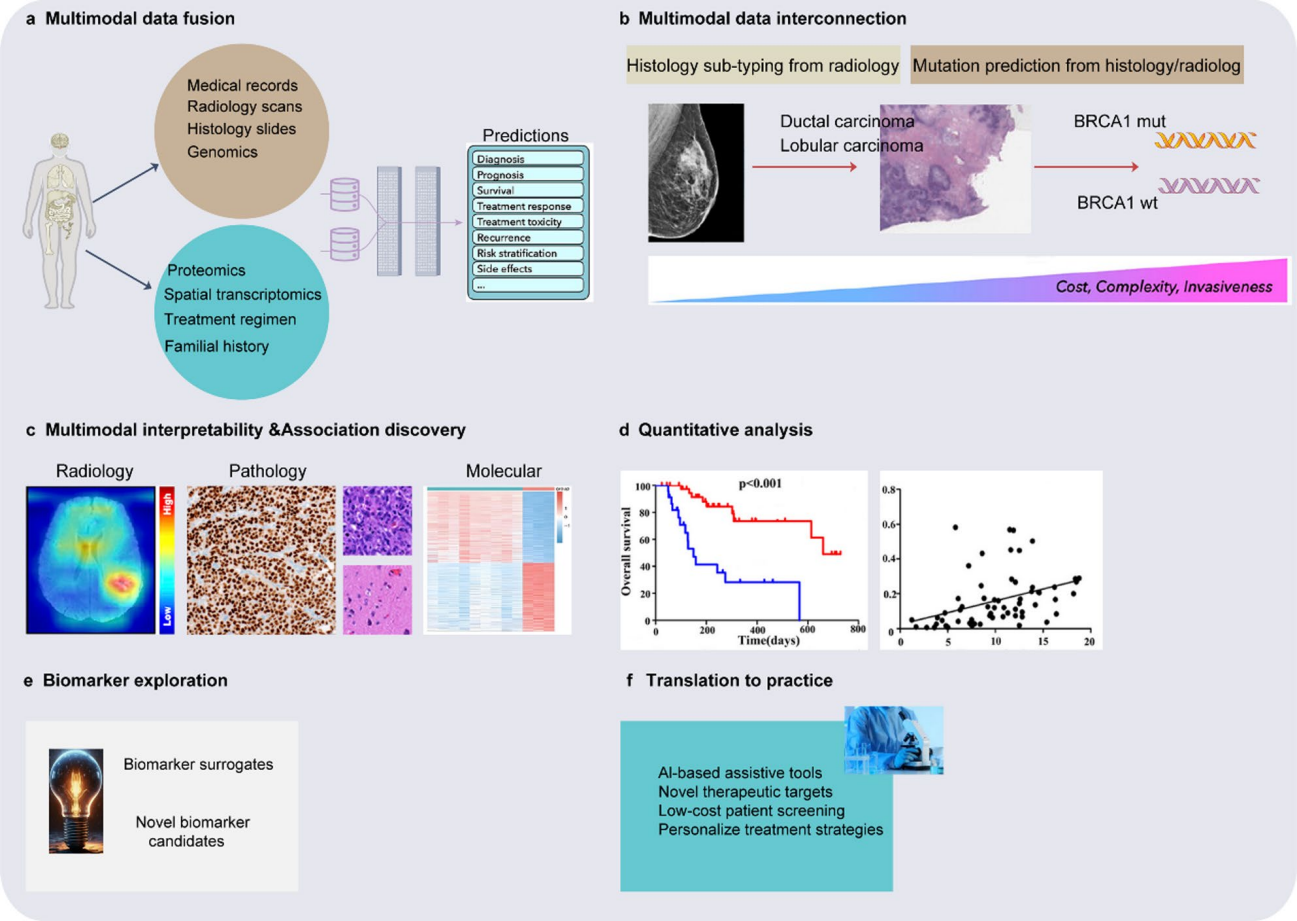
AI-driven multimodal data integration in oncology [9, 170–172]. (**a**) AI models can integrate complementary information and clinical context from diverse data sources to enhance the accuracy of outcome predictions. The clinical insights derived from these models can be further explored using interpretability methods and quantitative analysis (**c-d**), facilitating the identification of novel biomarkers and therapeutic targets (**e-f**). (**b**) AI can uncover novel multimodal relationships, such as the link between specific mutations and cellular morphology changes or the associations between radiological findings and histological tumor subtypes or molecular characteristics. These insights can offer non-invasive or cost-effective alternatives to existing biomarkers, enabling large-scale patient screening (**e-f**)

The design and integration of multimodal diagnostic and therapeutic platforms is another key direction in the collaboration between AI and nanotechnology [72, 121–123]. Multimodal platforms combine various diagnostic and therapeutic technologies (Fig. 5), such as MRI, photoacoustic imaging, photodynamic therapy, and chemotherapy, with the aim of achieving more comprehensive tumor management [84, 115, 124–128]. However, the integration of these technologies faces significant challenges, including the optimization of material properties, the synthesis of multifunctional nanoparticles, and the coordination of diagnostic and therapeutic parameters. AI technology plays a crucial role in supporting multimodal platforms by integrating data from different modalities to generate more precise diagnostic and treatment plans. AI can simultaneously analyze MRI and photoacoustic imaging data, combining the spatial distribution and functional characteristics of tumors to optimize the selection of treatment areas and dosage distribution [127, 129–132]. Moreover, AI can predict the synergistic effects of different treatment methods, such as the combined mechanism of photodynamic therapy and chemotherapy, guiding the functional design of multimodal platforms [72]. The research team led by João Conde was the first to evaluate the therapeutic efficacy of nanoparticles in cancer treatment using a machine-learning framework. Random forest analysis combined with SHAP revealed that the treatment modality itself—particularly photothermal therapy—plays a more decisive role in therapeutic outcomes than the intrinsic physicochemical properties of nanoparticles, with integrated photothermal therapy plus photoacoustic imaging showing the strongest advantage as a theranostic strategy [92]. LDA further indicated that shape may be more critical than size, with rod-shaped nanoparticles outperforming spherical ones, while surface chemistry, charge, and functionalization level emerged as key determinants of efficacy [92]. Regarding treatment strategies, photothermal and photodynamic therapies generally outperformed chemotherapy, and multimodal combinations showed synergistic benefits; conversely, multiple dosing correlated with poorer tumor reduction, often reflecting treatment-resistant tumors [92]. Although local and systemic administration showed comparable efficacy, current studies overwhelmingly favor systemic delivery (>75%). Therefore, more systematic investigation of locally administered nanoparticles is needed to optimize dosing strategies and nanomedicine design [92].

In terms of technical integration, AI helps optimize the structure and functionalization strategies of nanomaterials by simulating the in vivo behavior of nanoparticles, ensuring the efficient collaboration of different modal functions [133, 134]. This AI-driven multimodal diagnostic and therapeutic platform, which integrates the advantages of precise diagnosis and multifunctional therapy, provides innovative solutions to overcome the challenges of treating complex tumors, and is expected to further advance the intelligent and personalized development of cancer diagnosis and treatment in the future.

## Advantages and challenges of converging technologies

### Significant advantages of convergence technology

The integration of AI and nanotechnology has significantly enhanced the efficiency of cancer treatment, pioneering faster and more precise therapeutic models. The application of nanotechnology in cancer treatment relies on the accuracy of material design and selection, while traditional material development often requires extensive experimental validation, which is time-consuming and costly. The introduction of AI has greatly accelerated this process. Machine learning-based algorithms can extract key features from vast material databases to predict the properties of novel nanomaterials [135], such as targeting delivery efficiency, drug release characteristics, and biocompatibility [136, 137]. This data-driven optimization strategy significantly reduces the time and resources invested in experimental screening. By using AI models to quickly screen nanoparticle carrier materials, researchers can design more targeted drug delivery systems, achieving more efficient targeted therapy [138]. Additionally, AI integrates genomic, transcriptomic, and metabolomic data from patients to generate personalized treatment plans [139]. This treatment design, which incorporates the patient’s biological characteristics, not only optimizes therapeutic strategies but also significantly increases the success rate of cancer treatments. The remarkable advantages of this integrated technology make the entire process—from material selection to treatment plan generation—more efficient, bringing revolutionary changes to cancer therapy.

The integration of technologies has also significantly improved the patient experience by reducing toxic side effects and enhancing treatment predictability, making the treatment process safer and more comfortable. Nanotechnology can reduce drug distribution in non-target tissues by targeting drug delivery, thereby lowering toxicity [140–144]. However, relying solely on physicochemical design is insufficient to completely solve this issue. The involvement of AI further improves the targeting ability and drug release control accuracy of nanomaterials [85]. AI models can design nanoparticle carriers that respond under specific conditions (such as changes in acidity or enzyme concentration) based on the characteristics of the patient’s tumor microenvironment, ensuring that the drug is released only at the tumor site [145, 146]. This precise control significantly reduces systemic side effects during treatment. Furthermore, AI can monitor the patient’s treatment response and biological indicators in real-time, predicting potential side effects or deviations in efficacy, and adjusting the treatment strategy accordingly [147–149]. This real-time regulation capability not only reduces patient discomfort during treatment but also boosts treatment confidence. This advantage of integrated technology reflects patient-centered innovative thinking, which not only enhances treatment outcomes but also greatly improves the patient’s quality of life.

### Key challenges for the convergence of AI and nanotechnology

With the continuous development and integration of artificial intelligence (AI) and nanotechnology, both fields have demonstrated immense potential across various domains, including healthcare, pharmaceuticals, and environmental monitoring. However, despite the broad prospects this integrated technology offers for future technological innovation, its application still faces numerous challenges. The first challenge lies in data acquisition and sharing. The integration of AI and nanotechnology often requires vast, complex, and diverse data support, which typically originates from various fields such as biology, chemistry, and clinical medicine [150–153]. These data sources not only differ in format and standards but also in data collection, storage, and analysis methods, creating significant barriers to interdisciplinary collaboration. Nanotechnology applications in biomedicine require precise nanoscale data, while AI needs large volumes of medical imaging, clinical experimental data, and patient feedback, among other information. The key to advancing the integration of AI and nanotechnology is effectively consolidating these data from different sources and formats, and enabling their sharing across interdisciplinary teams. However, the current standardization process for data across different disciplines is lagging, and mechanisms for data sharing between institutions have not yet formed universal standards, hindering the collaborative innovation of AI and nanotechnology. Therefore, it is essential to establish unified global data standards, promote the interoperability and sharing of data across fields, and foster the integration and development of AI and nanotechnology.

Safety and ethical issues represent another significant challenge in the integration of AI and nanotechnology. The application of AI in medical diagnostics, drug development, and other areas is increasingly widespread, but the transparency of its decision-making process remains insufficient. AI algorithms often rely on complex deep learning models, the workings of which are often opaque to users, leading to the “black box” problem. In the medical field, AI decisions can influence patient health and even life or death. If the decision-making process of AI algorithms is not transparent and patients and doctors cannot understand the underlying logic, AI judgments may be questioned or rejected [74, 154, 155]. Moreover, the application of nanotechnology in drug delivery, cancer treatment, and other areas still faces potential safety risks [156]. For example, the behavior of nanoparticles in the body may be influenced by numerous factors, such as particle size, surface properties, and interactions with the biological system [157, 158]. These unknown factors could lead to unintended biological effects or even toxic reactions. Therefore, in the process of integrating AI and nanotechnology, ensuring the transparency of the technology, enhancing the interpretability of algorithms, and conducting rigorous safety assessments to mitigate potential risks are crucial for realizing its clinical applications. To address these issues, researchers and developers need to take additional measures to ensure that AI systems’ decision-making processes are traceable and to strengthen the evaluation and regulation of nanotechnology safety, ensuring that the technology meets ethical standards in its applications.

Finally, cost and accessibility are also significant challenges faced by the integration of AI and nanotechnology [159]. Although AI and nanotechnology hold vast application potential in many fields, the research, development, and implementation of these technologies often require substantial financial support. In low-resource environments, ensuring that these high-tech innovations are accessible to a broader population becomes a bottleneck limiting their widespread application. Nanotechnology itself requires expensive materials and equipment, making its production cost high, while AI technology demands enormous computational resources, including powerful computing capabilities and high-end hardware. The high costs of these technologies make them difficult to spread in economically disadvantaged regions, leading to regional disparities in technological applications. Furthermore, the integration of AI and nanotechnology not only requires significant hardware investments but also demands many skilled professionals to operate and maintain the systems, which presents a shortage of talent in many low-income countries and regions. Although global technology companies and government agencies are increasing investment in these fields, how to achieve the widespread adoption of these technologies in low-resource settings remains a pressing issue. To enable the global application of AI and nanotechnology, it is necessary to reduce the cost of these technologies by optimizing production processes, lowering research and development costs, and innovating business models, while strengthening international collaboration to improve the accessibility of technology in low-resource areas through shared technological outcomes and experiences.

### Limitations and challenges in the clinical translation of AI-nanomedicine

Despite rapid progress in AI–nanomedicine research, its clinical translation remains hindered by several systemic barriers. First, the combined nature of “algorithms + nanocarriers” introduces uncertainty at the levels of data, models, and materials. Significant variations across centers—including imaging acquisition parameters, nanomedicine preparation workflows, and differences in animal and patient populations—can lead to performance drift when models are applied across platforms or cohorts. In addition, the highly opaque decision-making process of deep learning models makes it difficult to align their outputs with established mechanistic knowledge, undermining trust from clinicians and regulatory authorities. Second, current drug–device regulatory frameworks are designed primarily for traditional therapeutics or static algorithms and lack mature pathways for evaluating the risks of combination products involving “continuously updating models + novel nanocarriers.” How to balance continuous learning and adaptive parameter updating with safety and risk control remains an unresolved challenge in regulatory science.

At the same time, biocompatibility and long-term safety, ethical and societal implications, and system-level interoperability remain key constraints for the clinical implementation of AI–nanomedicine. Nanoparticles optimized by AI may deviate substantially from conventional design paradigms in terms of structure, surface chemistry, and degradation pathways. Their potential immunogenicity and chronic toxicity therefore require systematic evaluation through cross-scale toxicology and long-term in vivo tracking, supported by a tiered safety assessment framework spanning high-throughput in vitro screening, animal studies, and preclinical validation. Ethically and in terms of governance, algorithmic bias may reinforce or even exacerbate disparities in healthcare, while unclear responsibility and attribution mechanisms weaken trust from both clinicians and patients. This underscores the need for governance frameworks grounded in privacy-preserving computation, enhanced interpretability, and clearly delineated accountability. In addition, heterogeneity among imaging platforms, databases, and algorithmic architectures continues to hinder multicenter validation and reproducibility. To enable large-scale clinical deployment of AI–nanotechnology, it is essential to establish higher-level international standards and interoperability protocols, creating a secure, transparent, and auditable foundational environment that supports future clinical translation.

## Conclusion and perspective

The deep integration of AI and nanotechnology is revolutionizing the cancer diagnosis and treatment system. Traditional therapies such as surgery, radiotherapy, and chemotherapy, while effective to some extent, generally face limitations such as insufficient precision and noticeable side effects. AI, through deep learning and big data analysis, extracts key information from medical imaging, genomics, and clinical data, significantly improving the accuracy of early screening and the personalization of treatment plans. Nanotechnology, on the other hand, leverages functionalized nanoparticles to achieve precise drug delivery, increasing drug concentration at the tumor site while minimizing damage to normal tissues. The collaborative innovation between the two creates a positive feedback loop: AI optimizes algorithms and predictive models to guide the design and refinement of nanodrugs, enhancing their targeting, stability, and biocompatibility; nanotechnology, in turn, provides AI with high-resolution real-time data, further boosting the predictive capabilities of the models. This deep integration not only drives the development of cancer diagnosis and treatment toward precision, personalization, and intelligence, but also opens new pathways for medical research. Through AI’s rapid data analysis and the precise delivery of nanotechnology, the efficiency of cancer diagnosis, treatment, and monitoring is comprehensively improved. The accuracy of early screening and cure rates are also significantly enhanced, providing innovative solutions for the leapfrog development of precision medicine and personalized treatment. Below are the future development directions of AI and nanotechnology integration in the field of cancer diagnosis and treatment:

### Technology optimization and breakthroughs

In the process of integrating AI and nanotechnology, technological optimization is crucial. First, the intelligent development of AI algorithms forms the foundation for advancing this field. Currently, AI algorithms have made progress in medical image analysis and disease prediction, but their accuracy and flexibility still need improvement. In the future, AI must possess self-learning and optimization capabilities to better handle complex data, enhancing the accuracy of tumor prediction and drug response analysis. At the same time, nanotechnology requires the development of multifunctional, customized nanomaterials that possess precise targeting and drug release functions, and can switch between different treatment methods such as chemotherapy, gene therapy, and immunotherapy, offering personalized treatment plans for patients. Another key point is the establishment of an in vivo real-time data feedback and optimization mechanism. The targeted delivery and drug release of nanoparticles within the body should be continuously monitored and adjusted. AI algorithms can analyze the dynamic behavior of nanoparticles and optimize drug release rates to ensure optimal therapeutic effects. In the future, optimization mechanisms based on real-time data feedback will automatically adjust treatment plans according to the patient’s physiological status and tumor progression, improving treatment precision, reducing adverse reactions, and providing safer and more effective cancer treatment options.

### Exploration of emerging areas

Nanorobots combined with AI have shown immense potential in cancer treatment. Nanorobots can navigate precisely within the body and perform therapeutic tasks. When integrated with AI algorithms, they can accurately release drugs, repair cells, or intervene in cancer cells based on tumor characteristics, avoiding damage to normal tissues and optimizing the treatment path. This technology enhances treatment targeting and precision, reduces side effects of traditional methods, and improves patients’ quality of life. Additionally, the combination of AI and nanotechnology has also made breakthroughs in liquid biopsy. By enhancing the signals of cancer cells or circulating tumor DNA, AI algorithms can improve the accuracy of early screening, monitor disease progression in real-time, and formulate personalized treatment plans. The integration of photoacoustic imaging with therapeutic AI nanotechnology provides high-resolution imaging using optical and ultrasound waves. AI optimizes data analysis to accurately locate tumors and, combined with therapeutic techniques, guides the release of drugs or generates thermal effects from nanoparticles, thus enhancing treatment outcomes. This technology not only advances early diagnosis but also opens new pathways for cancer treatment.

### Framework for cross-cutting Cooperation

The key to advancing the integration of AI and nanotechnology lies in establishing a unified data platform and fostering interdisciplinary collaboration mechanisms. With the development of technology, the complex and diverse data require sharing and integration among researchers from different disciplines. Establishing a globally unified data platform must not only provide standardized formats but also feature good interoperability to support the flow and analysis of data across fields such as medicine, nanotechnology, and computer science. Interdisciplinary collaboration can bring together the expertise from various fields, drive technological breakthroughs, and accelerate the integration of AI and nanotechnology applications. Furthermore, the global application and equitable development of these technologies are crucial, especially in low-income and developing countries. By reducing costs, simplifying designs, and cultivating local talent, the technologies can be made accessible worldwide, enhancing global health levels and preventing the widening of the technology gap.

#### Reconstructing the regulatory and ethical framework for AI-nanomedicine

Despite significant progress in AI–nanomedicine research, its clinical translation remains constrained by complex regulatory and ethical challenges. As a hybrid system that integrates both “algorithmic intelligence” and “nanocarrier-based therapeutics,” it places unprecedented demands on safety, transparency, and reproducibility. Currently, regulatory agencies such as the U.S. FDA and the European EMA have not yet established unified guidelines for AI-driven nanoplatforms and are still exploring appropriate evaluation frameworks. Key challenges include validation pathways for adaptive algorithms, requirements for data quality and standardization, and long-term biosafety assessment of nanomaterials within intelligent drug-delivery systems. Furthermore, algorithm interpretability, model traceability, and compliance in cross-institutional data use are essential for achieving regulatory acceptance and building clinical trust. Moving forward, it is necessary to develop an interdisciplinary regulatory framework that integrates AI ethics, nanotoxicology, and data governance principles. Establishing AI regulatory sandboxes could allow controlled testing of algorithmic models and intelligent nanosystems to evaluate their safety and efficacy. Additionally, creating international multicenter evaluation networks and standardizing model interpretability will be crucial for enabling the safe, effective, and sustainable translation of AI–nanotechnology from laboratory research to clinical practice.

## Data Availability

No datasets were generated or analysed during the current study.

## Abbreviations

AI: Artificial intelligence
EPR: Enhanced permeability and retention
EVs: Extracellular vesicles
GAN: Generative adversarial network
GNN: Graph neural network
ICD: Immunogenic cell death
MRI: Magnetic resonance imaging
NPs: Nanoparticles
PDT: Photodynamic therapy
PD-L1: Programmed death-ligand 1
ROS: Reactive oxygen species
STING: Stimulator of interferon genes
TME: Tumor microenvironment
XAI: Explainable artificial intelligence
